# Risk factors modifying the double burden of malnutrition of young children in Thailand

**DOI:** 10.1111/mcn.12910

**Published:** 2020-06-30

**Authors:** Tomoo Okubo, Amynah Janmohamed, Chompoonut Topothai, Jessica L. Blankenship

**Affiliations:** ^1^ UNICEF Bangkok Thailand; ^2^ UNICEF East Asia & Pacific Regional Office Toronto Ontario Canada; ^3^ Department of Health Ministry of Public Health Nonthaburi Thailand; ^4^ UNICEF East Asia & Pacific Regional Office Bangkok Thailand

**Keywords:** child, double burden, malnutrition, Multiple Indicator Cluster Survey (MICS), Thailand

## Abstract

Thailand is now faced with a double burden of malnutrition. Using nationally representative data from the 2015–2016 Multiple Indicator Cluster Survey, we utilized multinomial logistic regression models to examine factors associated with stunting only, wasting only, overweight only, concurrent stunting and overweight, and concurrent stunting and wasting among children 0–59 months of age (*n* = 11,068). The prevalences of <5 stunting only (height‐for‐age *Z* score < −2 SD) and wasting only (WHZ < −2 SD) were 8.5% and 4.7%, respectively. The prevalence of <5 overweight only (WHZ > +2 SD) was 7.8%. Children 12–23 months (risk ratio [RR], 95% confidence interval [CI]: 1.47 [1.18, 1.83]; *p* < .01) and 24–35 months (RR, 95% CI: 1.56 [1.26, 1.94]; *p* < .001) were at increased risk for stunting only, compared with children 48–59 months. The strongest risk factor for stunting only was low birth weight (RR, 95% CI: 3.42 [2.86, 4.10]; *p* < .001). Children 0–5 months were at highest risk for wasting only, compared with children 48–59 months (RR, 95% CI: 2.91 [2.16, 3.92]; *p <* .001). Children 48–59 months and male children were more likely to be overweight only. Higher household wealth and smaller household size were also significant predictors of overweight only. A small proportion of children were concurrently stunted and overweight (1.3%) and concurrently stunted and wasted (0.6%). A multipronged approach focused on adequate prenatal care, improving breastfeeding and complementary feeding practices, and mitigating the growing burden of overweight is needed to address the double burden of malnutrition in Thailand.

**Key messages**

- As low birth weight is a leading driver of stunting in Thailand, policy interventions should target improving women's nutrition during the preconception and prenatal period.
- The risk of stunting only is significantly higher among children 12–35 months, implying a cumulative effect of growth faltering in utero and poor caring and feeding practices in early childhood.
- Infants 0–5 months have a much higher risk of wasting, compared with older infants and young children, and children 48–59 months and boys are at increased risk for overweight.
- Integrated interventions to improve the quality of breastfeeding and complementary feeding practices and to lower the consumption of obesogenic foods are needed to address the double burden of malnutrition in young children.
- Social protection programmes should address regional and socioeconomic disparities in the prevalence of both undernutrition and overweight in young children in Thailand.

## 1 INTRODUCTION

An increasing number of countries are experiencing concurrent burdens of undernutrition and overnutrition, posing immense costs to individuals and overstretched health systems. The 2018 Global Nutrition Report (Development Initiatives, 2018) estimates that ~30% of 141 countries have high coexisting burdens of child stunting, anemia among women of reproductive age, and female adult overweight, of which 13 are low‐income and 19 are lower middle income countries. Further, estimates based on data from 106 countries indicate that ~2% of children < 5 years (~8 million) are both stunted and overweight (United Nations Children's Fund, 2018). Although these population level data are of serious public health concern, less is known about their key drivers at the household and individual levels, knowledge that is essential for appropriate nutrition action at the country level.

Improvements in child health during recent years have resulted in substantial reductions in child mortality and aspects of undernutrition in Thailand. These health gains are attributed to the country's rapid social and economic development, with substantial declines in poverty and improved access to quality healthcare services (World Bank, 2019). The introduction of Thailand's universal healthcare scheme in 2001 has also been credited with contributing to the reductions in child morbidity and mortality in the country (World Health Organization [WHO], 2017).

However, recent data indicate that malnutrition remains a pressing child health concern in Thailand, with a 10.5% prevalence of stunting and a stagnated >5% prevalence of wasting among children under 5 years of age (Blankenship, 2015; National Statistical Office, 2016). Moreover, the prevalence of child overweight has steadily increased to 8% among children under 5 (Blankenship, 2015; National Statistical Office, 2016). Therefore, like other countries experiencing rapid economic growth and shifting dietary and lifestyle patterns, Thailand now faces a double burden of malnutrition in its youngest children.

An effective policy and programme response to addressing Thailand's nutrition challenges requires an understanding of the key drivers of the growing double burden of malnutrition in the country, which have not been established. In this study, we examined the key influencers of child stunting, wasting, and overweight in Thailand using a nationally representative sample of children under 5 years of age.

## 2 METHODS

We utilized data from the 2015–2016 Multiple Indicator Cluster Survey (MICS) conducted in all 77 provinces and regions. Household surveys were conducted during November 2015 to March 2016 and included a wide range of health, nutrition, and social indicators for children < 5 years of age and women and men 15–49 years of age. A complete description of the survey design and methodology is published elsewhere (National Statistical Office, 2016). Briefly, a multistage sampling design was used in which enumeration areas (clusters) were randomly selected from each of the country's five regions (Bangkok, Central, North, Northeast, South) using probability proportional to size sampling. In each cluster, 20 households were randomly selected for a total sample of 31,010 households.

The primary study outcomes were stunting only, wasting only, and overweight only in children 0–59 months of age. Stunting was defined as height‐for‐age *Z* score (HAZ) < −2 SD, wasting was defined as weight‐for‐height *Z* score (WHZ) < −2 SD, and overweight was defined as WHZ > +2 SD based on the WHO Child Growth Standards (WHO & UNICEF, 2009). For the analysis, children were categorized as stunted only, wasted only, overweight only, concurrently stunted and overweight, concurrently stunted and wasted, or normal. The two concurrent outcomes were assessed to determine the prevalence and associated risk factors for these coexisting states in the first 5 years of life. The household wealth index developed for the 2015–2016 MICS analysis (National Statistical Office, 2016) using principal components analysis was used to categorize households into wealth quintiles.

Bivariate analyses were conducted to examine factors associated with under‐five stunting only, wasting only, overweight only, concurrent stunting and overweight, and concurrent stunting and wasting including child age, sex, and birth weight, maternal age and education, household size, area, wealth status, and type of sanitation facility. Multivariate logistic regression models were created to examine predictors of the five outcomes, and collinearity was examined between all variables. A multinomial logistic regression model was created to examine predictors of stunting only, wasting only, and overweight only for children 0–59 months, as compared with children with normal nutrition status. Variables tested were based on the UNICEF conceptual framework for causes of malnutrition (United Nations Children's Fund, 2015) and included child feeding indicators of minimum dietary diversity (MDD), minimum meal frequency (MMF), consumption of iron‐rich foods, consumption of animal‐source foods, and consumption of vitamin A‐rich foods, receipt of ≥4 antenatal care (ANC) visits, first ANC visit in the first trimester, receipt of ANC from a skilled care provider, skilled provider delivery, prevalence of diarrhea, acute respiratory infection, and fever. Model covariates were selected based on a significant (*p* < .05) multivariate relationship. The model controlled for the effect of the cluster sampling design through use of SPSS Complex Samples (SPSS Version 22.0). Results are presented as adjusted relative risk ratios (RRR; 95% confidence interval [CI]) using a two‐sided significance level (α = .05). Analyses were conducted using SPSS Version 22.0 (Armonk, NY: IBM Corp).

## 3 RESULTS

A total of 9,399 households with 11,068 children 0–59 months were included in the survey (Table 1). The proportion of boys and girls was 51.3% (*n* = 5,678) and 48.7% (*n* = 5390), respectively. More than 40% (43.2%; *n* = 4,061) of households were located in urban areas and almost all households (97.4%; *n* = 9,150) had access to an improved sanitation facility. The largest proportion of households were in the Northeast Region (31.8%; *n* = 2,989), followed by South Region (29.4%; *n* = 2,764), and 4.4% (*n* =410) of households were located in Bangkok. The average age of children and mothers was 30.5 months and 23.2 years, respectively. Sixty percent of mothers (*n* = 5,858) had nine or more years of education. Of children 0–59 months included in the survey, 8.8% (*n* = 975) had a low weight at birth (<2,500 g; Table 1).

**Table 1:** Participant characteristics

	*n*	% (mean)
Child characteristics	11,068	
Child age (months)		
0–5	580	5.2
6–11	670	6.1
12–23	2,261	20.4
24–35	2,496	22.6
36–47	2,562	23.1
48–59	2,499	22.6
Child sex		
Female	5,390	48.7
Male	5,678	51.3
Low birth weight (<2,500 g)	975	8.8
Maternal characteristics	9,765	
Maternal age (years)	9,765	(23.2)
Maternal education		
< 9 years	3,907	40.0
≥ 9 years	5,858	60.0
Household characteristics	9,399	
Household size (# of members)	9,399	(4.9)
Region		
Central	1,452	15.4
North	1,784	19.0
Northeast	2,989	31.8
South	2,764	29.4
Bangkok	410	4.4
Area		
Rural	5,338	56.8
Urban	4,061	43.2
Wealth quintile^a^		
Poorest	2,236	23.8
Second	2,065	22.0
Third	1,965	20.9
Fourth	1,906	20.3
Wealthiest	1,227	13.0
Improved sanitation facility^b^	9,150	97.4

*Note*. The sample included 11,068 children, <5, 9,765 mothers, and 9,399 households. Only mothers of sampled children were included in the analysis (other caregivers excluded).

a Obtained from MICS 6 calculation: National Statistical Office (2016).

b Flush/pour flush to piped sewer system, septic tank, or pit latrine; ventilated improved pit latrine; pit latrine with slab; composting toilet.

Among children 0–59 months, the prevalence of being stunted only was 8.5%, with the highest prevalence among children 12–23 months (10.2%) and 24–35 months (10.9%) of age (Table 2). Boys had a higher prevalence of stunting only compared with girls (8.8% vs. 8.2%), and the highest prevalence of stunting only was in South Region (11.5%). The prevalence of wasting only among children 0–59 months was 4.7%, with a significantly higher prevalence in infants 0–5 months (11.5%), compared with 3.5–4.5% in the other age groups (*p* < .01; Table 2). Male infants 0–5 months of age had more than three times the prevalence of wasting only compared with other age groups (*p* < .01). Similar to stunting only, the highest prevalence of wasting only was in South Region (7.1%). The prevalence of overweight only among children 0–59 months was 7.8%, with the highest prevalence in children 24–35 months (11.3%) and 48–59 months (10.4%), in contrast to the lowest prevalence among children 0–5 months (1.2%; *p* for trend < .01; Table 2). Boys 48–59 months had a significantly higher prevalence of overweight only compared with girls (13.1% vs. 7.5%; *p* < .05). The highest prevalence of overweight only was in Bangkok (10%), with a significantly higher prevalence among boys, compared with girls, in this region (13.5% vs. 6.4%; *p* < .05). The prevalence of concurrent stunting and overweight was 1.3%, with a higher prevalence among boys, compared with girls, at 36–47 months (*p* < .01) and 48–59 months (*p* < .001; Table 2). The prevalence of concurrent stunting and wasting was <1% (Table 2).

**Table 2:** Bivariate analysis for stunting, wasting, overweight, concurrent stunting and overweight, and concurrent stunting and wasting by child age group, region, and household wealth quintile

		Stunted only^a^			Wasted only^b^			Overweight only^c^			Stunted and overweight^d^			Stunted and wasted^e^		
Variables	*n*	Male	Female	Total	Male	Female	Total	Male	Female	Total	Male	Female	Total	Male	Female	Total
Child age (months)					**		**	**	**	**	*	*				
0–5	580	6.9	8.5	7.7	14.7	8.1	11.5	0.7	1.8	1.2	4.2	1.5	2.9	1.8	0.0	0.9***
6–11	670	8.3	6.8	7.7	3.9	3.8	3.8	2.5	1.1	1.9	0.4	1.7	0.9*	0.1	0.0	0.0
12–23	2,261	10.4	9.9	10.2	3.1	5.1	4.0	6.2	5.8	6.0	0.6	1.3	0.9	1.2	0.1	0.7***
24–35	2,496	13.3	8.5	10.9	3.9	4.7	4.3	10.9	11.6	11.3	1.7	1.0	1.3	1.0	0.2	0.6**
36–47	2,562	5.6	8.1	6.8	2.9	4.1	3.5	8.5	8.8	8.6	3.2	0.5	1.9**	0.6	0.5	0.5
48–59	2,499	7.2	6.5	6.9	4.4	4.6	4.5	13.1	7.5	10.4*	1.1	0.1	0.6***	0.4	0.7	0.6
Region			*		**											
Central	1,718	11.3	7.7	9.7	4.4	4.5	4.5	5.8	8.8	7.2	2.6	1.1	1.9*	0.3	0.6	0.4
North	2,032	6.3	7.9	7.1	4.5	4.4	4.5	8.0	7.4	7.7	2.1	0.2	1.2***	1.3	0.3	0.8
Northeast	3,517	7.9	6.4	7.1	2.7	5.4	4.1*	9.7	7.0	8.3	0.9	1.1	1.0	1.0	0.1	0.5***
South	3,311	9.6	13.6	11.5	8.9	5.1	7.1*	7.8	6.6	7.2	1.7	0.9	1.3	0.7	0.5	0.6
Bangkok	490	7.0	5.9	6.5	1.9	3.9	2.9	13.5	6.4	10.0*	1.1	0.5	0.8	1.3	0.3	0.8
Household wealth quintile																
Poorest	2,619	10.8	8.9	9.9	3.6	6.8	5.2	7.5	5.8	6.7	2.1	0.7	1.4*	1.4	0.6	1.0
Second	2,425	9.1	8.6	8.8	4.0	5.7	4.8	8.1	5.8	7.0	2.1	0.6	1.4*	0.8	0.2	0.5**
Third	2,309	7.7	7.1	7.4	8.2	3.7	6.1	8.6	8.0	8.4	2.3	0.5	1.5**	0.4	0.7	0.5
Fourth	2,248	5.0	9.5	7.1	3.5	3.5	3.5	8.8	11.6	10.1	0.4	1.1	0.7	0.4	0.2	0.3
Wealthiest	1,467	12.9	5.9	9.5	3.3	3.9	3.6	7.8	5.8	6.8	2.0	1.5	1.8	1.1	0.0	0.6
Total	1,1068	8.8	8.2	8.5	4.5	4.9	4.7	8.2	7.4	7.8	1.8	0.8	1.3	0.8	0.3	0.6***

*Note*. Asterisks in male/female columns indicate significant differences by indicator category and asterisks in total columns indicate sex differences.

a Length/height‐for‐age < −2 SD below WHO Child Growth Standards median.

b Weight‐for‐length/height < −2 SD below WHO Child Growth Standards median.

c Weight‐for‐length/height > +2 SD above WHO Child Growth Standards median.

d Length/height‐for‐age < −2 SD below WHO Child Growth Standards median and weight‐for‐length/height > +2 SD above WHO Child Growth Standards median.

e Length/height‐for‐age < −2 SD below WHO Child Growth Standards median and weight‐for‐length/height < −2 SD below WHO Child Growth Standards median.

* *p* < .05,

** *p* < .01,

*** *p* < .001.

### 3.1 Influencing factors for stunting

Children 12–23 months (risk ratio [RR], 95% CI: 1.47 [1.18, 1.83]; *p* < .01) and 24–35 months (RR, 95% CI: 1.56 [1.26, 1.94]; *p* < .001) had the highest risk of being stunted only, as compared with children 48–59 months (Table 3). Children with low birth weight (<2,500 g) had a 3.4 times higher risk of stunting only (RR, 95% CI: 3.42 [2.86, 4.10]; *p* < .001), as compared with children born with normal weight. The risk of under‐five stunting only was positively associated with older maternal age (RR, 95% CI: 1.02 [1.01, 1.03]; *p* < .001). Children from households in the poorest wealth quintile had a higher risk of stunting only (RR, 95% CI: 1.44 [1.10, 1.88]; *p* < .01), compared with those in the wealthiest households. Having a non‐improved sanitation facility (RR, 95% CI: 1.64 [1.17, 2.30]; *p* < .01), larger household size (RR, 95% CI: 1.18 [1.14, 1.22]; *p* < .001), and maternal education less than 9 years (RR, 95% CI: 1.30 [1.10, 1.54]; *p* < .01) were also positively associated with child stunting only. Compared with Bangkok, only children living in the South Region had an increased risk of stunting only (RR, 95% CI: 1.63 [1.14, 2.34]; *p* < .01).

**Table 3:** Association between independent variables and stunting, wasting, overweight, concurrent stunting and overweight, and concurrent stunting and wasting among children 0–59 months

	Stunted only^a^		Wasted only^b^		Overweight only^c^		Stunted and overweight^d^		Stunted and wasted^e^	
	RRR	95% CI	RRR	95% CI	RRR	95% CI	RRR	95% CI	RRR	95% CI
Child age (months)										
0–5	0.97	0.72, 1.32	2.91***	2.16, 3.92	0.11***	0.06, 0.21	5.27***	2.70, 10.30	1.69	0.68, 4.17
6–11	0.89	0.65, 1.21	0.86	0.58, 1.29	0.16***	0.09, 0.26	1.67	0.70, 4.00	0.60	0.01, 1.58
12–23	1.47**	1.18, 1.83	0.95	0.71, 1.28	0.55***	0.44, 0.69	1.62	0.81, 3.24	1.27	0.60, 2.69
24–35	1.56***	1.26, 1.94	1.06	0.79, 1.41	1.14	0.94, 1.38	2.40**	1.26, 4.56	1.21	0.56, 2.58
36–47	0.97	0.76, 1.23	0.81	0.60, 1.10	0.80*	0.65, 0.97	3.08***	1.67, 5.70	0.90	0.41, 1.99
48–59 (Reference)	^—^	^—^	^—^	^—^	^—^	^—^	^—^	^—^	^—^	^—^
Child sex										
Female	0.87	0.76, 1.00	1.04	0.87, 1.25	0.86*	0.74, 0.99	0.50***	0.34, 0.71	0.38**	0.22, 0.66
Male (Reference)	^—^	^—^	^—^	^—^	^—^	^—^	^—^	^—^	^—^	^—^
Low birth weight (<2,500 g)	3.42***	2.86, 4.10	0.88	0.62, 1.24	0.81	0.60, 1.09	1.83*	1.13, 2.97	3.27***	1.78, 6.02
Maternal age (years)	1.02***	1.01, 1.03	1.03***	1.02, 1.04	1.00	0.99, 1.00	1.03***	1.02, 1.05	1.01	0.99, 1.03
Maternal education (<9 years)	1.30 **	1.10, 1.54	1.30*	1.06, 1.61	0.79*	0.65, 0.96	1.23	0.81, 1.85	1.57	0.96, 2.56
Region										
Central	1.38	0.99, 1.94	1.22	0.75, 1.99	0.73*	0.55, 0.99	3.23*	1.33, 7.84	0.43	0.15, 1.24
North	0.90	0.62, 1.31	1.02	0.60, 1.72	0.85	0.61, 1.18	1.88	0.72, 4.94	0.59	0.20, 1.79
Northeast	0.91	0.63, 1.31	0.92	0.55, 1.55	0.90	0.66, 1.23	2.09	0.80, 5.48	0.34	0.11, 1.05
South	1.63**	1.14, 2.34	1.71*	1.03, 2.85	0.78	0.56, 1.08	2.24	0.86, 5.85	0.53	0.17, 1.67
Bangkok (Reference)	^—^	^—^	^—^	^—^	^—^	^—^	^—^	^—^	^—^	^—^
Household area										
Urban	0.89	0.76, 1.05	0.76*	0.61, 0.94	0.98	0.83, 1.15	1.06	0.73, 1.54	0.89	0.49, 1.61
Rural (Reference)	^—^	^—^	^—^	^—^	^—^	^—^	^—^	^—^	^—^	^—^
Household wealth quintile										
Poorest	1.44**	1.10, 1.88	1.96***	1.35, 2.85	0.97	0.73, 1.31	0.82	0.43, 1.57	2.30	0.90, 5.92
Second	1.13	0.88, 1.45	1.47*	1.04, 2.10	1.10	0.84, 1.45	0.84	0.48, 1.49	1.09	0.42, 2.81
Third	0.88	0.68, 1.13	1.76**	1.25, 2.47	1.29	0.99, 1.68	0.89	0.52, 1.54	1.09	0.43, 2.76
Fourth	0.80	0.63, 1.02	0.99	0.69, 1.40	1.60***	1.25, 2.04	0.43**	0.23, 0.81	0.61	0.22, 1.67
Wealthiest (Reference)	—	^—^	^—^	^—^	^—^	^—^	^—^	^—^	^—^	^—^
Household size (# of members)	1.18***	1.14, 1.22	1.02	0.98, 1.08	0.96*	0.92, 0.99	0.85**	0.76, 0.94	1.16*	1.04, 1.31	
Unimproved sanitation ^g^	1.64**	1.17, 2.30	0.41*	0.20, 0.82	1.20	0.80, 1.78	4.41***	2.54, 7.65	0.53	0.11, 2.63	

*Note*. Continuous variables: maternal age and household size; categorical variables: child age group, child sex, low birth weight, maternal education, region, household area, household wealth quintile, and unimproved sanitation.

Abbreviations: CI, confidence interval; RRR, relative risk ratio.

a Length/height‐for‐age < −2 SD below WHO Child Growth Standards median.

b Weight‐for‐length/height < −2 SD below WHO Child Growth Standards median.

Weight‐for‐length/height > +2 SD above WHO Child Growth Standards median.

Length/height‐for‐age < −2 SD below WHO Child Growth Standards median and weight‐for‐length/height > +2 SD above WHO Child Growth Standards median.

Length/height‐for‐age < −2 SD below WHO Child Growth Standards median and weight‐for‐length/height < −2 SD below WHO Child Growth Standards median.

Flush/pour flush to elsewhere (not into a pit, septic tank, or sewer); pit latrine without slab/open pit; bucket toilet; hanging toilet/latrine; no facility/bush/field (open defecation).

* *p* < .05,

** *p* < .01,

*** *p* < .001.

### 3.2 Influencing factors for wasting

Infants 0–5 months were 2.9 times more likely to be wasted only (RR, 95% CI: 2.91 [2.16, 3.92]; *p* < .001), compared with children 48–59 months of age (Table 3). Children living in the poorest households (RR, 95% CI: 1.96 [1.35, 2.85]; *p* < .001), second quintile (RR, 95% CI: 1.47 [1.04, 2.10]; *p* < .05), and third quintile (RR, 95% CI: 1.76 [1.25, 2.47]; *p* < .01) were more likely to be wasted only, as compared with children in the wealthiest households. Similar to stunting only, children in South Region (RR, 95% CI: 1.71 [1.03, 2.85]; *p* < .05), older maternal age (RR, 95% CI: 1.03 [1.02, 1.04]; *p* < .001), and those whose mothers had fewer than 9 years of education (RR, 95% CI: 1.30 [1.06, 1.61]; *p* < .05) had a higher risk of wasting only. Living in urban areas (RR, 95% CI: 0.76 [0.61, 0.94]; *p* < .05) and having unimproved sanitation (RR, 95% CI: 0.41 [0.20, 0.82]; *p* < .05) were associated with a lower risk of wasting only.

### 3.3 Influencing factors for overweight

The risk of overweight only was lowest in the youngest children 0–5 months (RR, 95% CI: 0.11 [0.06, 0.21]; *p* < .001), 6–11 months (RR, 95% CI: 0.16 [0.09, 0.26]; *p* < .001), and 12–23 months (RR, 95% CI: 0.55 [0.44, 0.69]; *p* <0.001), compared with children 48–59 months of age (Table 3). Girls were less likely to be overweight only, compared with boys (RR, 95% CI: 0.86 [0.74, 0.99]; *p* < .05). Greater household wealth, maternal education > 9 years, and smaller household size were positively associated with child overweight only. Children in Central Region had a significantly lower risk of being overweight only (RR, 95% CI: 0.73 [0.55, 0.99]; *p* < .05), compared with those living in households in Bangkok, with no significant differences observed for the other regions.

Girls were less likely to be concurrently stunted and overweight, as compared with boys (RR, 95% CI: 0.50 [0.34, 0.71]; *p* < .001), and children with low birth weight were more likely to be concurrently stunted and overweight (RR, 95% CI: 1.83 [1.13, 2.97]; *p* < .05), as compared with those with birth weight ≥ 2,500 g. This was also observed for concurrent stunting and wasting, with a lower risk for girls (RR, 95% CI: 0.38 [0.22, 0.66]; *p* < .01) and a much higher risk for low birth weight children (RR, 95% CI: 3.27 [1.78, 6.02]; *p* < .001).

## 4 DISCUSSION

Our study revealed age‐specific determinants of undernutrition in children in Thailand, with infants 0–5 months having the highest prevalence of wasting only and children 24–35 months with the highest prevalence of stunting only. The very high percentage (~12%) of wasting among young infants, with nearly double the prevalence in boys compared with girls aged 0–5 months, is consistent with other evidence indicating a higher risk of wasting during the first 6 months of life where poor breastfeeding practices, low maternal body mass index, and inadequate weight gain during pregnancy are public health concerns (Kerac et al., 2011). The high burden of wasting in these youngest children is of serious concern, given the adverse impact of wasting on cognitive development at this stage (Prado & Dewey, 2014). The observed association between a non‐improved sanitation facility and reduced risk of child wasting in our study was unexpected. Although we are unable to explain this finding, it could likely be due to a combination of wasting being highest in the youngest infants, unimproved sanitation prevalence being low in the sample, and wasting prevalence not being higher in the poorest households, but rather uniformly elevated across wealth strata.

Low birth weight was a major driver of under‐five stunting in our study, with a 3.4 times higher risk of stunting among children born with low weight (<2,500 g), as compared with normal‐weight births. This finding is consistent with an earlier analysis of the determinants of child stunting in Thailand using 2012 MICS data that revealed that mothers' perceived large size of their child at birth was associated with a lower risk of stunting, compared with perceived small size at birth (Cetthakrikul et al., 2018). Despite nearly universal coverage of ANC in Thailand, low birth weight continues to be a problem, with an estimated prevalence of 9.4% (National Statistical Office, 2016), an increase from 8.0% in 2012 (National Statistical Office, 2012). Therefore, good maternal nutrition and adequate care during pregnancy are policy imperatives for the country. Although locally produced Triferdine, a prenatal supplement containing iron, iodine, and folic acid, is freely available through the public health system, evidence suggests that compliance is suboptimal due to fears of side effects and delivery complications (Sanghvi, Harvey, & Wainwright, 2010; Winichagoon, 2013). Efforts to promote regular consumption of a prenatal multiple micronutrient supplement should also be strengthened through health facility and community‐based counseling and behavior change communication strategies to improve maternal micronutrient status and birth outcomes (Haider & Bhutta, 2017; Kawai, Spiegelman, Shankar, & Fawzi, 2011).

In our study, stunting continued to increase in children 12–35 months. This is consistent with evidence from other low‐ and middle‐income countries (LMIC) indicating sustained growth faltering during the period of complementary feeding, with a progressive increase up to 24 months, after which a leveling off has been observed (de Onis & Branca, 2016). The prevalence of age‐appropriate breastfeeding for children < 2 years is only 28% in Thailand, with only one third of children receiving breastmilk at 12 months of age (National Statistical Office, 2016). Moreover, the prevalences of early initiation of breastfeeding (40%) and exclusive breastfeeding for 6 months (23%) are very low (National Statistical Office, 2016). Although dietary quality in Thailand is relatively high for the Asia‐Pacific region, with 75% and 85% of children 6–23 months achieving MDD and MMF, respectively, when accounted together, only 56% of children < 2 years receive a minimum acceptable diet (National Statistical Office, 2016). The similarity in rates of MDD, MMF, and minimum acceptable diet across geographic areas and socioeconomic strata in Thailand suggests that factors such as social norms and caregiver awareness may be influencing children's diets, apart from overall food availability.

A multipronged approach that includes large‐scale social and behavior change communication is recommended to increase awareness of age‐appropriate breastfeeding and complementary feeding practices and to promote greater consumption of micronutrient‐rich foods and properly formulated fortified foods (e.g., fortified infant cereals). In addition to awareness‐raising efforts, actions should be taken to reduce harmful marketing of breastmilk substitutes, a widespread practice in Thailand (Baker et al., 2016; Thepha, Marais, Bell, & Muangpin, 2018).

As expected, children in poorer households were more likely to be undernourished, whereas children in higher wealth households were at increased risk for overweight. However, it is important to note that the prevalence of overweight was elevated (7–10%) for all wealth quintiles. The propensity towards Western‐influenced diets and lifestyle patterns, with greater consumption of increasingly affordable energy‐dense processed foods and snacks high in fat, sugar, and salt, including lower quality diets in poor urban households, along with reduced physical activity, is becoming increasingly evident across all sociodemographic groups in LMICs like Thailand that are undergoing a nutrition transition (Pirgon & Aslan, 2015; Popkin, Adair, & Ng, 2012; Sahoo et al., 2015). To address the rising burden and early onset of overweight in Thailand, currently estimated at 8.2% among children < 5 years (National Statistical Office, 2016), national level policies that reduce marketing of “empty calorie” foods should be a mainstay of nutrition programming in the country.

In Thailand, addressing poverty‐related factors affecting child nutrition requires needs‐based social assistance to complement the country's universal health coverage system. Such “social safety net” programmes can effectively improve the nutritional status of women and children through enabling greater consumption of higher quality and diverse foods, better child care, and increased access to health services (Bastagli et al., 2016; Delisle & Batal, 2016). In 2015, the Thai government introduced the Child Support Grant (CSG) to reduce poverty and socioeconomic inequity and improve child health and well‐being in the early years of life. Although it originally started as a scheme to target poor families with children < 1 year of age, the government approved expansion of the CSG in 2019, extending critical support to children < 6 years from poor families with an annual income below 100,000 Baht (~USD 3,300; United Nations Children's Fund, 2019). A recent national impact evaluation of the CSG revealed improvements in health and nutrition indicators, including the incidence of wasting (Economic Policy and Research Institute, UNICEF, & Thailand Development Research Institute, 2019). Although the programme targets children at the age they are most vulnerable to and affected by the consequences of nutritional deprivation, expanding CSGs to include pregnant women, as well as leveraging the platform to provide nutrition‐specific interventions (e.g., education and micronutrient supplements), should be considered.

Our findings indicate that geographic factors are associated with the nutritional status of children in Thailand. The risks of stunting and wasting were higher among children in South Region, which includes the southernmost provinces that have experienced lagging economic development and civil unrest in recent years (Sondergaard et al., 2016). The implications of geographical disparities in nutritional status are particularly relevant for Thailand, given its highly centralized policy and administrative structures. Although improving the nutrition of women and children is a key focus of the country's National Economic and Social Development Plan (National Economic and Social Development Board, 2017), programme strengthening is also necessary at the subnational level with targeted measures for poor households to reduce regional health and nutrition inequities. Moreover, differences in malnutrition prevalence by province and ethnic group warrant further investigation to better understand contextual factors posing barriers to good maternal and child nutrition to inform policy directives for greater health and nutrition equity.

We explored predictors of stunting, wasting, and overweight in a large nationally representative sample of children under 5 years. In our analysis, we were not able to assess the effects of key factors known to be associated with child nutrition such as maternal anthropometry, household food security, and indicators for micronutrient status, water quality, and infection, as these data were not available in the MICS data set. Although indicators for infant and young child feeding practices such as exclusive breastfeeding, MDD, and MMF were collected in the survey, these data were available for a subset of children and were, therefore, excluded from our analysis. It should also be noted that MDD and MMF are population level indicators for children 6–23 months of age and are thus not representative of children's diets at the individual level. As diet‐related drivers of malnutrition are age‐dependent and the indicators are age‐specific, a more in‐depth analysis of children's diets and the association with nutritional status is necessary in Thailand to better understand prevailing risk factors for the double burden of malnutrition across different age groups in the country. Finally, our findings should be considered with the limitations of cross‐sectional surveys to infer causation and the potential for residual confounding.

Grappling with persistent undernutrition and the rapid emergence of overweight and related conditions poses additional challenges for already overburdened health sectors in LMICs. Understanding the complex interplay of factors driving the coexistence of multiple forms of malnutrition at all levels is important for effective risk reduction measures. The growing double burden of malnutrition among children in Thailand is of serious public health concern, given the potentially lifelong consequences for physical and cognitive impairment, and it also has substantial economic implications. Reducing malnutrition in the early years of life is one of the most cost‐effective investments for developing human capital, poverty reduction, and economic growth (Horton & Hoddinott, 2014). In Thailand, the estimated cost of stunting to the country's gross domestic product is 5% per capita, or approximately USD 20 billion (Galasso, Wagstaff, Naudeau, & Shekar, 2016). Moreover, an increasing prevalence of overweight has implications for higher rates of chronic illness and rising healthcare costs. A multisystem approach is needed, with policies informed by evidence of risk factors for undernutrition and overnutrition starting in early childhood. This should include interventions implemented through the health, food, WASH and social protection systems focused on creating adequate provision of and access to prenatal care, improving breastfeeding and complementary feeding practices, and mitigating the growing burden of overweight, alongside efforts to identify and address nutrition inequities.

## CONFLICTS OF INTEREST

The authors declare that they have no conflict of interest.

## CONTRIBUTIONS

TO and JLB designed the study; TO and JLB conducted the data analysis; AJ and JLB interpreted the data; TO drafted the manuscript; TO, AJ, CT, and JLB contributed to the preparation of the manuscript. All authors approved the final manuscript. TO and JLB had primary responsibility for the final content. The manuscript's contents are solely the responsibility of the authors and do not necessarily represent the official views of UNICEF or the Ministry of Public Health in Thailand.
